# Early Life Manipulations of the Nonapeptide System Alter Pair Maintenance Behaviors and Neural Activity in Adult Male Zebra Finches

**DOI:** 10.3389/fnbeh.2016.00058

**Published:** 2016-03-29

**Authors:** Nicole M. Baran, Michelle L. Tomaszycki, Elizabeth Adkins-Regan

**Affiliations:** ^1^Department of Psychology, Cornell UniversityIthaca, NY, USA; ^2^School of Biology, Georgia Institute of TechnologyAtlanta, GA, USA; ^3^Department of Psychology, Wayne State UniversityDetroit, MI, USA; ^4^Department of Psychology, Lafayette CollegeEaston, PA, USA; ^5^Department of Neurobiology and Behavior, Cornell UniversityIthaca, NY, USA

**Keywords:** vasopressin, nonapeptides, V1aR, development, zebra finch, pair bonding

## Abstract

Adult zebra finches (*T. guttata*) form socially monogamous pair bonds characterized by proximity, vocal communication, and contact behaviors. In this experiment, we tested whether manipulations of the nonapeptide hormone arginine vasotocin (AVT, avian homolog of vasopressin) and the V1a receptor (V1aR) early in life altered species-typical pairing behavior in adult zebra finches of both sexes. Although there was no effect of treatment on the tendency to pair in either sex, males in different treatments exhibited profoundly different profiles of pair maintenance behavior. Following a brief separation, AVT-treated males were highly affiliative with their female partner but sang very little compared to Controls. In contrast, males treated with a V1aR antagonist sang significantly less than Controls, but did not differ in affiliation. These effects on behavior in males were also reflected in changes in the expression of V1aR and immediate early gene activity in three brain regions known to be involved in pairing behavior in birds: the medial amygdala, medial bed nucleus of the stria terminalis, and the lateral septum. AVT males had higher V1aR expression in the medial amygdala than both Control and antagonist-treated males and immediate early gene activity of V1aR neurons in the medial amygdala was positively correlated with affiliation. Antagonist treated males showed decreased activity in the medial amygdala. In addition, there was a negative correlation between the activity of V1aR cells in the medial bed nucleus of the stria terminalis and singing. Treatment also affected the expression of V1aR and activity in the lateral septum, but this was not correlated with any behaviors measured. These results provide evidence that AVT and V1aR play developmental roles in specific pair maintenance behaviors and the neural substrate underlying these behaviors in a bird.

## Introduction

The nonapeptides—oxytocin (OT) and arginine vasopressin (AVP) in mammals; mesotocin (MT) and arginine vasotocin (AVT) in birds—are important modulators of social behaviors, including pair bonding and affiliation, across a wide range of vertebrate taxa (Insel and Young, 2001; Goodson, 2005; Choleris et al., 2013). However, comparatively little is known about the role played by nonapeptide hormones during the development of the social brain (Cushing, 2013).

Zebra finches (*Taeniopygia guttata*) establish life-long pair relationships and both parents participate in the care of the young. Additionally, zebra finches exhibit a shift in their social preferences, from attachment to parents early in life to a pair relationship with an opposite sex conspecific in adulthood (Immelmann, 1972; Zann, 1996). Both of these relationships are characterized by constant physical proximity, frequent vocal communication, and affiliative contact behaviors, such as clumping (perching in contact), allopreening (mutual grooming), and spending time together inside of a nest (Zann, 1996).

In recent years, several studies have provided support for the hypothesis that the nonapeptides play an important role in pair bonding and affiliative behaviors in birds. These studies have also identified several key nodes of the social behavior network which are involved in affiliation and pairing, including the paraventricular nucleus of the hypothalamus (PVN), the medial amygdala (MeA, or the nucleus taeniae of the amygdala in birds), the medial bed nucleus of the stria terminalis (BSTm), and the lateral septum (LS; Newman, 1999; Goodson, 2005; Kelly and Goodson, 2014). However, research from both rodents and zebra finches suggests that courtship, pair formation, and pair maintenance are each differentially regulated by multiple brain regions and neuromodulators (Aragona et al., 2006; Prior and Soma, 2015).

Unlike findings from socially monogamous rodents, partner preference is not induced by central infusions of either AVT or MT in adult zebra finches (Goodson et al., 2004). Nevertheless, it is clear that nonapeptides are important in the formation and maintenance of affiliative relationships in zebra finches. Pairing for 48 h increases expression of both AVT and MT in both the PVN and the BSTm in both sexes (Lowrey and Tomaszycki, 2014). Consistent with this finding, experimental knockdown of endogenous MT production in the PVN increases the latency to pair in females and reduces affiliative behaviors in zebra finches of both sexes (Kelly and Goodson, 2014). Furthermore, antagonists acting primarily at the VT3 (OT-like) receptor increases the latency to pair and decreases pair formation in zebra finches (Pedersen and Tomaszycki, 2012; Klatt and Goodson, 2013). Knockdown of AVT production in the PVN and BSTm cell groups and infusion of AVT antagonists into the lateral septum (LS) also reduces social flocking in both sexes (Kelly et al., 2011; Kelly and Goodson, 2014). Additionally, contact behavior with pair partners is correlated with immediate early gene activity in the MeA in females (Svec et al., 2009).

Despite evidence that nonapeptides play an important role in mediating social relationships across taxa in adulthood, there is little work on their role in development. Manipulations of the AVP system during development can affect social behaviors in both juvenile and adult rats (Boer, 1985; Winslow and Insel, 1993; Boer et al., 1994; Veenema et al., 2012; Bredewold et al., 2014). In socially monogamous prairie voles (*Microtus ochragaster*), OT or OT antagonist injections on postnatal day one impact both pair formation and partner preference, and lead to alterations in nonapeptide receptor binding in adults of both sexes (Bales and Carter, 2003a,b; Yamamoto et al., 2004; Bales et al., 2007).

We previously demonstrated in zebra finches that nonapeptide system manipulations early in life altered patterns of affiliative interest in both parents and opposite sex conspecifics throughout development (Baran et al., 2016). Thus, we predicted that these effects would extend into adult pairing relationships.

In this experiment, we tested the hypothesis that AVT and a nonapeptide antagonist exert developmental effects on the neural pathways underlying species-typical affiliative behavior in zebra finches. We manipulated the nonapeptide system of zebra finch chicks on days 2–8 post-hatch via daily intracranial (IC) injections of either AVT, Manning Compound (MC, a potent vasopressin 1a receptor antagonist) or saline (vehicle control) and assessed the effects on pairing behaviors in adulthood, the expression of vasotocin 1a receptor (V1aR), and the immunoreactivity of the immediate early gene, ZENK (Egr-1, a marker of neural activation) in three brain regions—the MeA, BSTm, and LS. We predicted that injections of AVT early in development would lead to more rapid pair formation relative to Controls, an increase in affiliation with the pair partner, and increased V1aR expression and ZENK immunoreactivity. We predicted that MC would have the opposite effect, decreasing V1aR expression and affiliative behaviors.

## Materials and methods

### Breeding and housing conditions

Experimental subjects were raised in the breeding colony at Cornell University. As juveniles, subjects were cared for by the parents, which were provided with *ad libitum* access to finch seed, cuttlebone, grit, water, and supplemented weekly with hard-boiled egg. After 40–45 days post-hatch, subjects were removed from their natal aviary and housed in same-sex aviaries in a separate room from the parents. Each same-sex aviary contained birds of the same treatment to control for possible social interactions between birds in different treatments. The present study used the same subjects as Baran et al. (2016).

### Intracranial injections

Chicks of each sex were randomly assigned to a treatment group on day 2 post-hatch, following genetic sexing. From day 2 through day 8 post-hatch, subjects received daily 2 μL intracranial injections of either (1) AVT (10 ng, (Arg8)-Vasotocin, Bachem 1785.0005); (2) Manning Compound (MC), a potent V1a and mild OT receptor antagonist (50 ng, d(CH2)51,Tyr(Me)2,Arg8)-Vasopressin, Bachem 5350.0005); or (3) 0.9% isotonic saline (Manning et al., 1989; Goodson et al., 2004). AVT and MC act at multiple receptor subtypes in the zebra finch brain, including the VT4 (V1aR), VT3 (OT-like), and V2 receptors (Manning et al., 2012; Busnelli et al., 2013). IC injections were performed by hand using a sterile 31G insulin syringe, similar to Bender and Veney (2008) and see also Baran et al. (2016). The chicks behaved normally immediately following injections, including normal begging and locomotor behavior, and there was no increase in mortality associated with treatment. All procedures were developed with veterinary supervision and approved by Cornell University's Institutional Animal Care and Use Committee (Protocol # 2011-0130).

### Pairing

Subjects were randomly assigned an unmanipulated pair partner of the opposite sex (hereafter “partner,” regardless of pair status) on day 90 post-hatch. Partners were drawn from a population of unpaired birds 180–365 days old. All partners had been housed in same-sex aviaries and rooms since 45–50 days post-hatch and thus were sexually-naive and unpaired.

Introductions (Day 1) between the subjects and their pair partner occurred in a small aviary (57 × 32 × 42 cm) in a room with no other birds. After the introductions (lasting 15–45 min), the pair was moved into a pair aviary in a colony room. Each aviary (57 × 32 × 42 cm or 61 × 36 × 43 cm) was provided with *ad libitum* access to finch seed, cuttlebone, grit, and water. To avoid the potentially confounding effects of breeding attempts, pairs were not provided access to either nesting material or nest boxes. Pairs were visually, but not acoustically, separated from other pairs.

Pairs were filmed in their home aviaries for 16 min on each of days 2, 3, 4, and 6 following introduction between 12:00 and 18:00. All videos were scored by a trained coder who was blind to treatment for the duration of time spent perched in contact (clumping) and allopreening (both by the subject and by their partner). To test for the effects of AVT treatment on both general activity level and on water balance, we also measured the time spent with the head in the food dispenser and the number of visits to both the food dispenser and water dispenser.

### Mate separation, reunion, and euthanasia

On the seventh day following introduction, subjects were separated briefly from their pair partner. The partner was removed from the pair aviary for 1 h and moved temporarily to a same-sex social aviary located in another room. Previous studies have shown that a brief separation induces a stress response and leads to an increase in affiliative behaviors upon reunion (Remage-Healey et al., 2003; Prior et al., 2013, 2014). After 1 h of separation, the partner was returned to the pair aviary and the reunion was filmed for 25 min. Videos were scored for the same measures as above, plus the number of song bouts performed by male subjects.

Following an additional 55–65 min (90 min post-reunion) in the pair cage which was not filmed, the subjects were euthanized via rapid decapitation. Brains were immediately extracted, frozen in cold methylbutane, and stored at −80°C. Brains were sectioned coronally into six series at 20 μm and mounted onto charged slides. The number of birds that completed the study are as follows: Control males (*N* = 7); AVT males (*N* = 11); MC males (*N* = 11); Control females (*N* = 10); AVT females (*N* = 9); and MC females (*N* = 9).

### Probe preparation

We developed primers from published sequences (Genbank: V1aR = XM_002187285) using the NCBI primer tool (Forward = AGCGCGGCTCGCAAGTCTAC; Reverse = GAAGGGCGCCCAGCAAACGA). We conducted reverse-transcribed PCR (Life Technologies: #12574-035) per manufacturer's instructions on isolated RNA to obtain the cDNA and sequenced the product at the Applied Genomics Technology Center (AGTC) at Wayne State University. We prepared the probe using a DIG RNA Labeling kit according to manufacturer's instructions (Roche Applied Science: # 11175025910).

### Immunocytochemistry and *in situ* hybridization

Fluorescence immunocytochemistry and *in situ* hybridization were conducted using a protocol adapted from previous work (Lowrey and Tomaszycki, 2014). Slides were fixed with 3% paraformaldehyde, acetylated, dehydrated, and air dried. Hybridization with a probe concentration of 1:1000 occurred at 55°C overnight. After a series of washes, slides were incubated in 0.3% hydrogen peroxide in Tris-NaCl-Tween (TNT) buffer for 10 min, and blocked in TNT buffer with 2 mg/ml of bovine serum albumin for 30 min. Slides were then incubated in the secondary antibody (1:100, Anti-DIG-POD, Roche Applied Science: #11207733910) for 2 h, followed by 30 min in a tyramide-conjugated fluorophore (1:100, Alexa 488, Life Technologies: # T-20922). Slides were then processed for ZENK immunocytochemistry. Each step was preceded by 3 washes (5 min each) in Tris-buffered saline (TBS). Slides were blocked in 2% normal goat serum and 0.3% Triton X-100 for 30 min. This was followed by a 48 h incubation in the ZENK primary antibody (1:1000, Santa Cruz Biotechnology: # sc189) at 4°C. Slides were then incubated for 2 h in the secondary antibody (5 μl/ml; goat anti-rabbit secondary conjugated to Alexa 594, Life Technologies: # A-11012). Finally, slides were cover-slipped with Slow Fade Antifade Gold with DAPI (Life Technologies: #S36938) and sealed. V1aR sense slides exhibited no staining, and staining in the anti-sense slides appeared to have a distribution similar to that reported in earlier studies (Leung et al., 2011). All slides were stained at the same time, to control for potential differences in staining quality.

### Quantification

Slides were analyzed using a Leica DM 5500B microscope. We quantified the number of cells in a 400 X 400 μm counting frame for BSTm and MeA and 200 X 400 μm counting frame for LS (by hand, using the Image J Cell Counter plugin to keep track of cell counts). For ZENK-ir, red cells exhibiting a punctate nuclear-associated staining were quantified. We counted the number of DAPI+ nuclei using the particle analysis in Image J. For the MeA, 4 ± 2 counting frames taken from 3 separate sections (one anterior, one intermediate, and one posterior) representing both left and right hemispheres were quantified per animal. For the BSTm, 3 ± 1 counting frames taken from 2 separate sections [one ventral to the anterior commissure (AC) where the AC is strongest and the second where the AC just begins to disappear and the BSTm cells appear to spill over the occipito-mesencephalic tract (OM)]. For LS, 4 ± 2 counting frames taken from 3 separate sections (one anterior, one intermediate, and one posterior). Counts from the LS were scaled by a factor of two to be comparable to the other two regions.

For each counting frame, 3 measurements were taken: the number of cells expressing V1aR, the number of cells immunoreactive for ZENK (ZENK-ir), and the number of cells expressing both. Due to tissue quality, 1 Control male, 2 AVT males, and 4 MC males were excluded from the analysis. The final sample size was 6 Control males, 9 AVT males, and 7 MC males.

### Statistical analysis

All statistical analyses were performed with R software (R Core Team, 2015). To test if treatment affected whether or not an individual formed a pair, we used a general linear model (GLM) with a binomial link function. To test the effect of the treatment on the amount of clumping, allopreening, time in the food dispenser, visits to the food dispenser, and visits to the water dispenser across test days, we used a random slope linear mixed model (LMM). In this model, Sex, Treatment, and Test Day (Day 2, 3, 4, 6, and Reunion) were specified as fixed factors. Random factors were individual ID nested within Family ID. The interaction effect considered was Sex*Treatment*Test. We used a Poisson GLM to test the effect of treatment on the number of song bouts performed by males during the reunion.

To analyze cell count data, we averaged the cell counts within regions and within subjects and rounded the counts to the nearest integer. We then analyzed the counts using weighted negative binomial GLMs using the *glm.nb* function in the MASS package, with the number of sections contributing to the averaged count included as the weight. Poisson GLMs are preferable to log-transforming count data (O'Hara and Kotze, 2010). Negative binomial regression is a generalization of Poisson regression, but it has an extra parameter to model over-dispersion. We used weights to ensure that counts obtained from a larger number of sections were weighted more in the analyses, since we can be more confident that these counts represent the true mean within a given individual and brain region.

First, we tested whether there was an effect of treatment on the expression of V1aR, ZENK-ir, V1aR+ZENK cells, and the number of DAPI-stained nuclei within the MeA, BSTm, and LS. Second, we tested for associations between these neural measures and behavior, including treatment as a factor in the model. To perform model comparisons for the GLM, LMM, and negative binomial GLM models, we used likelihood ratio tests to compare the full model to a reduced null model with the factor of interest removed using the *anova* function to perform a chi-square test. In addition, we performed *post-hoc* tests using the *testInteractions* function in the phia package. All data are available in Data Sheet 1.

## Results

### Pairing behavior

There was no effect of treatment in either sex on whether subjects formed a pair [GLM: Males, *X*^2^(2) = 4.2, *p* = 0.1; Females, *X*^2^(2) = 1.6, *p* = 0.4] nor on the number of days until the first observed instance of either perching in contact or allopreening [Log-rank test: Males: *X*^2^(2) = 2.6, *p* = 0.3; Females: *X*^2^(2) = 0.7, *p* = 0.71]. Consistent with previous studies, both male and female subjects spent more time either perched in contact or allopreening during the reunion compared to observations on previous days, controlling for both sex and treatment [LMM, Clumping, *X*^2^(4) = 19.5, *p* = 0.0006; Allopreening, *X*^2^(4) = 27.6, *p* < 0.0001]. This demonstrates that our separation and reunion paradigm reliably elicited pair maintenance behaviors.

**Figure 1:**
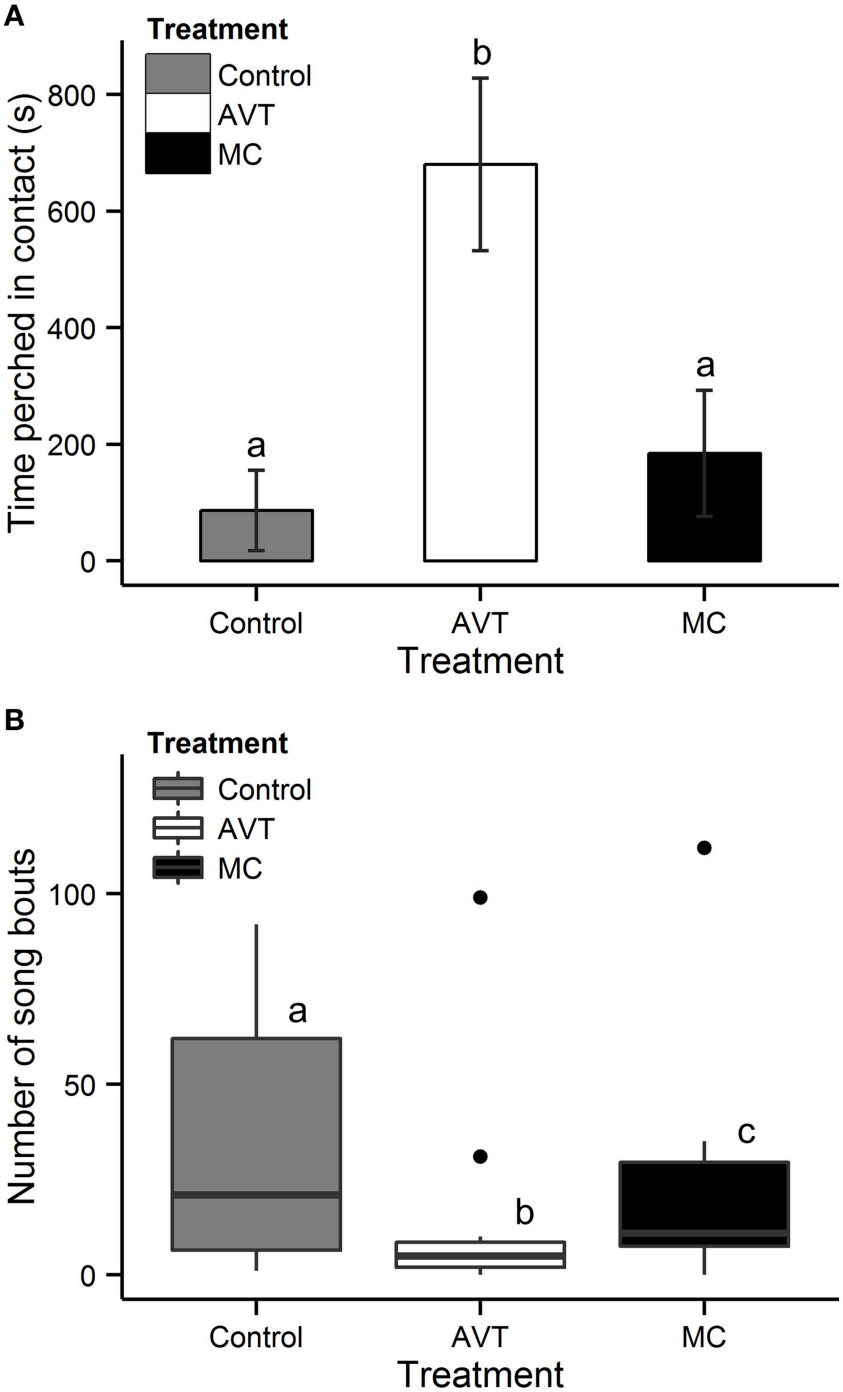
**Time perched in contact and number of song bouts by male subjects. (A)** Mean ± SE time (s) spent perched in contact with the female partner during the 25 min reunion period following a 1 h separation. **(B)** Box-and-whisker plot of the number of song bouts during the first 25 min following reunion with the female partner after a 1 h separation. Data beyond the ends of the whiskers are outliers and plotted as points. Subjects received intracranial injections on post-hatch days 2–8 of either arginine vasotocin (AVT), Manning Compound (MC, a V1aR antagonist), or vehicle control of saline (Control). Letters indicate groups that are significantly different from each other.

In males, there was a significant effect of treatment on affiliative behavior during the 25 min reunion period [LMM, *X*^2^(2) = 15.7, *p* = 0.0004; Figure 1A, Supplementary Video]. AVT males spent, on average, 45% of the 25 min reunion period perched in contact with their female partner, which was 790% more than Control males [*X*^2^(1) = 17.5, *p* < 0.0001] and 360% more than MC males [*X*^2^(1) = 14.0, *p* = 0.0004]. MC and Control males did not differ from each other in the time spent clumping [*X*^2^(1) = 0.4, *p* = 0.5]. Treatment did not affect the amount of time females spent perched in contact with their male partner [LMM, *X*^2^(2) = 2.2, *p* = 0.3].

We also observed a significant effect of treatment on the number of song bouts performed by subject males during the reunion period [Poisson GLM: *X*^2^(2) = 80.1, *p* < 0.0001; Figure 1B]. Control males sang the greatest number of song bouts and AVT males sang the fewest (Control-AVT: *Z* = −8.7, *p* < 0.0001; Control-MC: *Z* = −3.2, *p* = 0.003; and AVT-MC: Z = 5.7, *p* < 0.0001). Typically, AVT males sang a small number of directed songs upon reunion with the female, whereas both Control and MC males sang throughout the entire reunion period.

### Feeding and drinking behavior

Treatment did not alter the proportion of time spent in the food dish or number of visits to the food dish, controlling for both sex and test day [Proportion: *X*^2^(2) = 0.1, *p* = 0.9; Number of visits: *X*^2^(2) = 2.6, *p* = 0.3]. There was also no significant effect of treatment on the number of drinks of water consumed by subjects across test days [*X*^2^(2) = 4.5, *p* = 0.1].

### Effect of treatment on V1aR expression and the immediate early gene ZENK

**Figure 2:**
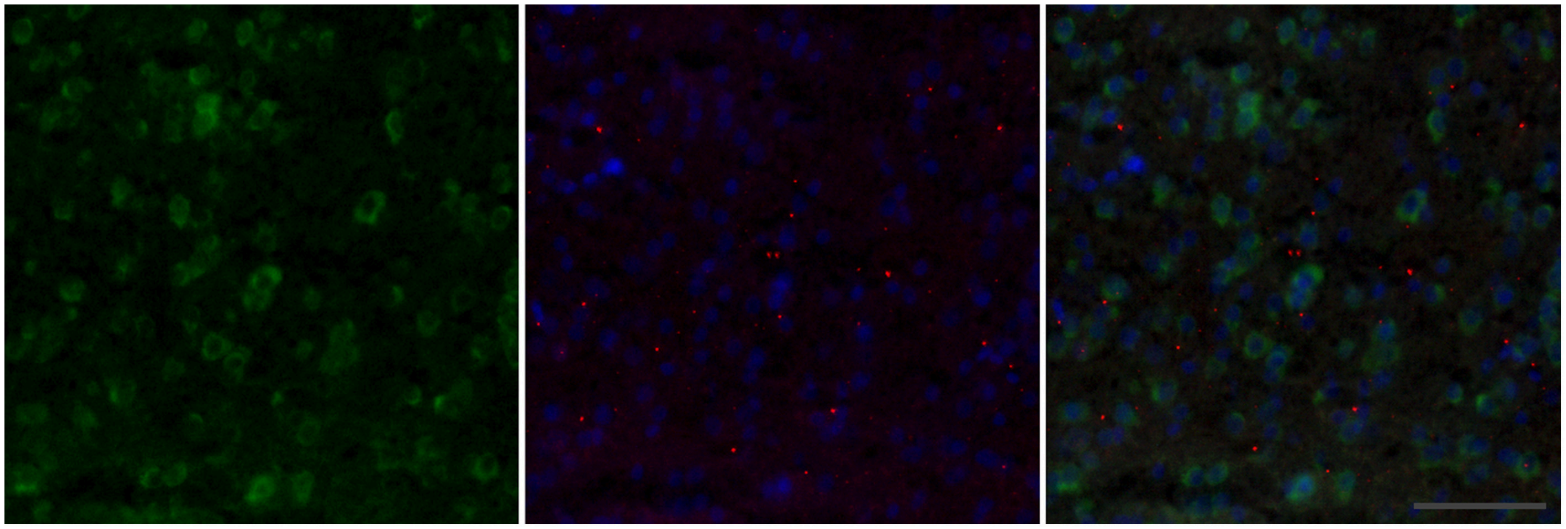
**Example double-label fluorescence *in situ* staining of the BSTm of a Control male**. In the first panel, V1aR expressing cells are labeled in green. In the second panel, punctate nuclear-staining for ZENK is shown in red, with DAPI nuclear stain shown in blue. The third panel shows all three—V1aR, ZENK, and DAPI—combined. The frame is 200 × 200 μm and the scale bar is 50 μm.

We next examined the effects of the intracranial injections on the expression of V1aR and immediate early gene activity in the BSTm, LS, and MeA. Because we only found treatment effects on behavior in males, we restricted our analyses to male subjects. Across all treatment groups, there were more V1aR-expressing cells per 1600 μm^2^ counting frame in the BSTm (190 ± 61 cells) and the LS (204 ± 85 cells) compared to the MeA (82 ± 33 cells) [nbGLM: *X*^2^(2) = 265.3 *p* < 0.0001]. In addition, there were more ZENK-ir cells in the LS compared to the other two regions (BSTm: 31 ± 24; LS: 48 ± 23; MeA: 31 ± 18) [nbGLM: *X*^2^(2) = 48.7, *p* < 0.0001; Figure 2].

**Figure 3:**
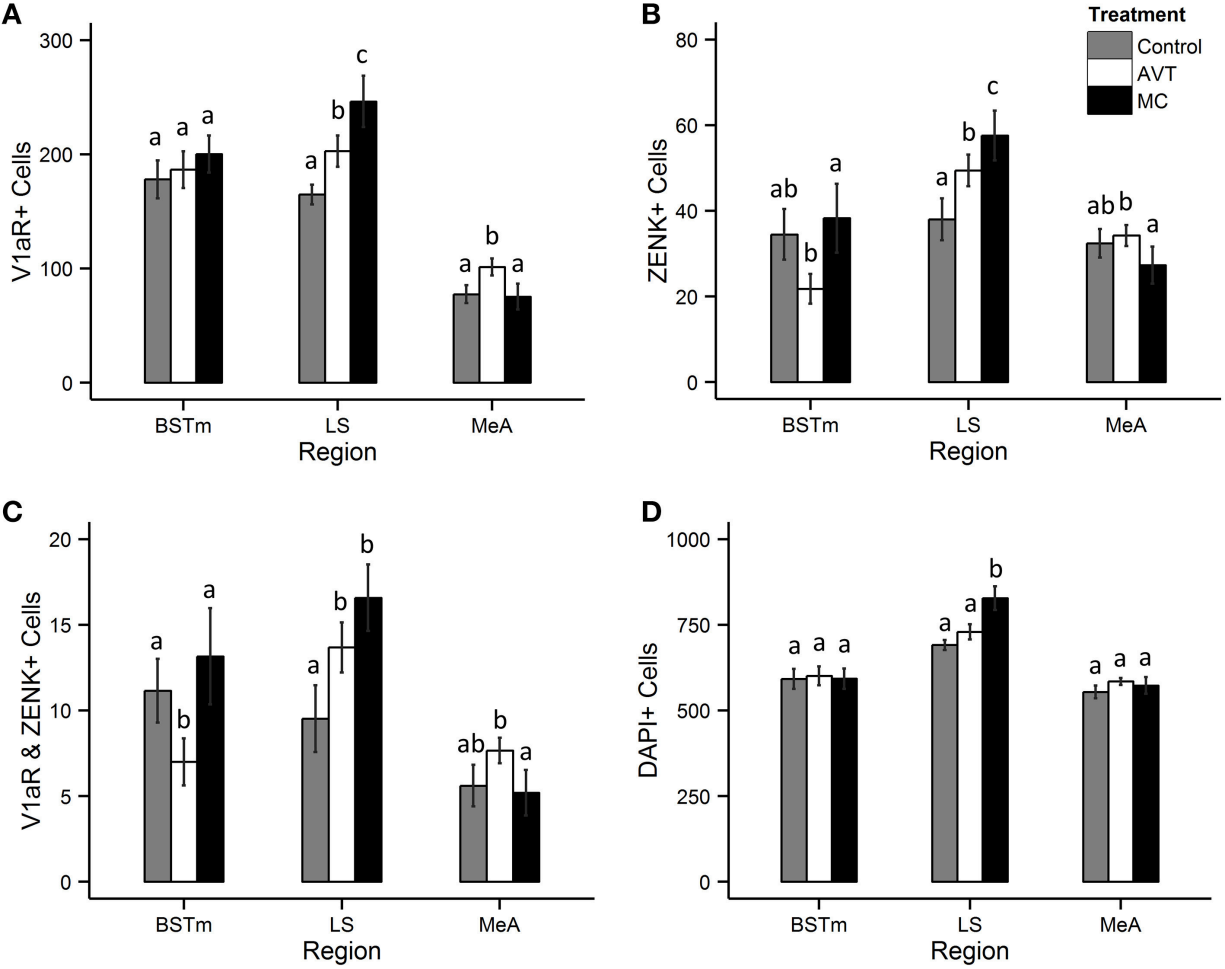
**Effects of treatment on the number of V1aR and ZENK expressing cells in the BSTm, LS, and MeA. (A)** Mean ± SE number of cells expressing V1aR, **(B)** Mean ± SE number of cells immunopositive for ZENK (Egr-1), **(C)** Mean ± SE number of cells expressing both V1aR and ZENK, and **(D)** Mean ± SE DAPI-stained nuclei in the bed nucleus of the stria terminalis (BSTm), lateral septum (LS), and the medial amygdala (MeA) in a 1600 μm^2^ counting frame. Letters indicate groups that are significantly different from each other.

Treatment was a significant predictor of the number of V1aR-expressing cells in both the MeA [nbGLM: *X*^2^(2) = 34.6, *p* < 0.0001] and LS [nbGLM: *X*^2^(2) = 27.9, *p* < 0.0001], but not the BSTm [nbGLM: *X*^2^(2) = 1.9, *p* = 0.4; Figure 3A]. In the MeA, AVT subjects had more V1aR-expressing cells compared to the other groups [Control-AVT: *X*^2^(1) = 21.9, *p* < 0.0001; Control-MC: *X*^2^(1) = 0.2, *p* = 0.6; AVT-MC: *X*^2^(1) = 32.8, *p* < 0.0001]. In the LS, both MC and AVT subjects had more V1aR expressing cells, with MC having the most [Control-AVT: *X*^2^(1) = 9.1, *p* = 0.005; Control-MC: *X*^2^(1) = 33.7, *p* < 0.0001; AVT-MC: *X*^2^(1) = 8.0, *p* < 0.005].

Treatment was also a significant predictor of ZENK-ir in all three brain regions [nbGLM, BSTm: *X*^2^(2) = 11.8, *p* = 0.003; LS: *X*^2^(2) = 27.3, *p* < 0.0001; MeA: *X*^2^(2) = 5.8, *p* = 0.05; Figure 3B]. MC males had fewer ZENK-ir cells in the BSTm compared to AVT males [Control-AVT: *X*^2^(1) = 6.5, *p* = 0.02; Control-MC: *X*^2^(1) = 0.3, *p* = 0.6; AVT-MC: *X*^2^(1) = 11.7, *p* = 0.002]. In the LS, MC males had the most ZENK-ir cells, followed by AVT males, with Control males having the fewest [Control-AVT: *X*^2^(1) = 13.4, *p* = 0.0005; Control-MC: *X*^2^(1) = 32.5, *p* < 0.0001; AVT-MC: *X*^2^(1) = 4.4, *p* = 0.04]. AVT males were found to have fewer ZENK-ir cells in the MeA compared to MC males [Control-AVT: *X*^2^(1) = 0.1, *p* = 0.7; Control-MC: *X*^2^(1) = 2.8, *p* = 0.18; AVT-MC: *X*^2^(1) = 5.8, *p* = 0.05].

Treatment also predicted the co-localization of V1aR and ZENK in all three brain regions [nbGLM, BSTm: *X*^2^(2) = 12.4, *p* = 0.002; LS: *X*^2^(2) = 12.8, *p* = 0.001; MeA: *X*^2^(2) = 8.1, *p* = 0.02; Figure 3C]. In the BSTm, AVT males had fewer V1aR+ZENK cells compared to both MC and Control males [Control-AVT: *X*^2^(1) = 5.7, *p* = 0.03; Control-MC: *X*^2^(1) = 0.7, *p* = 0.4; AVT-MC: *X*^2^(1) = 13.1, *p* = 0.0009]. In the LS, both MC and AVT males had more V1aR+ZENK cells than Controls [Control-AVT: *X*^2^(1) = 5.7, *p* = 0.03; Control-MC: *X*^2^(1) = 13.9, *p* = 0.0006; AVT-MC: *X*^2^(1) = 1.9, *p* = 0.17]. In the MeA, AVT males had more V1aR+ZENK cells than MC males [Control-AVT: *X*^2^(1) = 3.4, *p* = 0.1; Control-MC: *X*^2^(1) = 0.3, *p* = 0.5; AVT-MC: *X*^2^(1) = 7.2, *p* = 0.02].

There was no significant effect of treatment on the total number of cells per counting frame (i.e., the number of DAPI-stained nuclei) in the extended medial amygdala [nbGLM, BSTm: *X*^2^(2) = 0.4, *p* = 0.8; MeA: *X*^2^(2) = 5.4, *p* = 0.07; Figure 3D]. This suggests that treatment did not impact the overall density of cells in these regions. However, treatment did impact the number of DAPI stained nuclei in the LS [nbGLM: *X*^2^(2) = 16.8, *p* = 0.0002], with MC males having more DAPI stained nuclei in the LS compared to Control and AVT males [Control-AVT: *X*^2^(1) = 1.6, *p* = 0.2; Control-MC: *X*^2^(1) = 17.8, *p* < 0.0001; AVT-MC: *X*^2^(1) = 8.7, *p* = 0.006].

### Correlations between behavior, immediate early gene activity, and V1aR

ZENK-ir in the MeA was positively correlated with the time the subject spent perched in contact with his female partner [nbGLM: *X*^2^(1) = 5.6, *p* = 0.02]. We also observed a significant negative correlation between singing and ZENK-ir in the BSTm [nbGLM: *X*^2^(1) = 5.7, *p* = 0.02]. There were no correlations observed between ZENK-ir and either clumping or singing in the LS.

**Figure 4:**
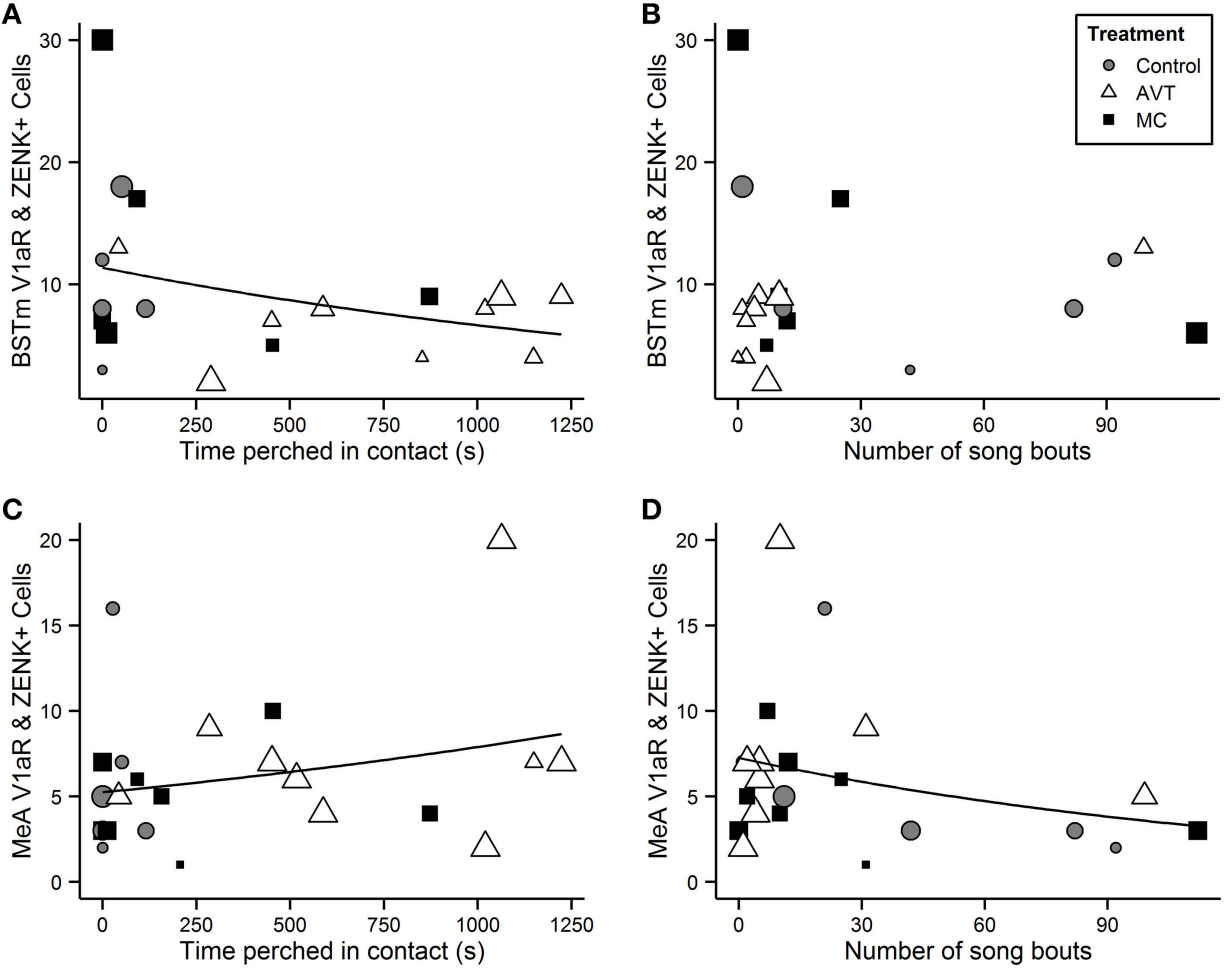
**Number of V1aR+ZENK cells in the BSTm and MeA in relation to clumping and singing**. Scatterplot of the number of cells per 1600 μm^2^ counting frame in the bed nucleus of the stria terminalis (BSTm, **A,B**) and medial amygdala (MeA, **C,D**) expressing both V1aR and ZENK in relation to time (s) spent perched in contact (clumping) with the female partner **(A,C)** and the number of song bouts during the reunion period following a 1 h separation **(B,D)**. The size of the points is scaled to the number of counting frames per subject, from the largest (*n* = 4 for BSTm and *n* = 6 for MeA and LS) to the smallest (*n* = 1). The lines depict significant Poisson general linear model fits.

The number of V1aR+ZENK cells in the extended medial amygdala (BSTm and MeA) was correlated with behaviors exhibited by males during the reunion. In the BSTm, there was a negative relationship between the number of V1aR+ZENK cells and clumping behavior [nbGLM: *X*^2^(1) = 8.0, *p* = 0.005; Figure 4A], but was no significant relationship between V1aR+ZENK co-localization and singing behavior [nbGLM: *X*^2^(1) = 1.4, *p* = 0.2; Figure 4B]. In the MeA, there was a strong positive relationship between V1aR+ZENK co-localization and clumping behavior [nbGLM: *X*^2^(1) = 11.5, *p* = 0.0007; Figure 4C] and a strong negative relationship between V1aR+ZENK co-localization and the number of song bouts [nbGLM: *X*^2^(1) = 9.9, *p* = 0.002; Figure 4D]. There was no relationship between V1aR+ZENK co-localization in the LS and behavior [nbGLM, Clumping: *X*^2^(1) = 0.016, *p* = 0.90; Singing: *X*^2^(2) = 0.005, *p* = 0.94].

## Discussion

To our knowledge, these results are the first demonstration that the nonapeptide system may function early in development to influence adult social behaviors in birds, similar to what has been observed in prairie voles (Stribley and Carter, 1999; Bales and Carter, 2003a,b; Yamamoto et al., 2004). Males in each of the three treatment groups exhibited dramatically different profiles in their pair maintenance behaviors, and yet they all appeared to form pair bonds. AVT males were highly affiliative, perching in contact with their partner eight times as much as Control males. AVT males also had higher expression levels of V1aR and more cells which co-localized both V1aR and ZENK in the MeA. MC males, on the other hand, were as affiliative as Controls, but sang far less. MC males also had reduced ZENK-ir in the BSTm and more V1aR+ZENK cells in the LS. We also demonstrated that co-localization of V1aR expression and ZENK-ir in the MeA and BSTm is correlated with both affiliation and amount of singing.

These findings are broadly consistent with the now accumulating evidence that nonapeptides play an important role in pair bonding in birds. However, they are the first results to demonstrate that AVT and the V1aR receptor are important in pairing behavior in male zebra finches. Previous research on the role of nonapeptides in pairing in zebra finches found significant effects of MT and OT-like receptors, mostly in females, but had not identified a role for V1aR in pairing in either sex (Goodson et al., 2004; Pedersen and Tomaszycki, 2012; Klatt and Goodson, 2013; Kelly and Goodson, 2014). The present findings are thus consistent with decades of research in prairie voles demonstrating that AVT and V1aR are important for pairing in males (Winslow et al., 1993; Pitkow et al., 2001). It should be noted, however, that one possible explanation for the lack of observed effects in females is that the intracranial injections occurred before functional receptors were expressed at high enough levels in females to have obvious developmental effects on brain and behavior. In rodents, the development of the AVT system of females is delayed relative to males and a similar phenomenon may be occurring in birds as well (Buijs et al., 1980; Szot and Dorsa, 1993).

Although previous studies have provided only modest evidence of V1aR expression in the MeA, LS, or BSTm in zebra finches (Leung et al., 2009, 2011), we consistently observed staining in these regions. V1aR expression is relatively low, but highly variable (15.1 ± 6.8% of neurons in the MeA, 26.9 ± 7.8% in the LS, and 30.6 ± 12.0% in the BSTm). All of these regions do express this receptor subtype in prairie voles and there is evidence of AVT binding within these regions in zebra finches, white throated sparrows, and canaries (Voorhuis et al., 1988; Leung et al., 2009, 2011). Thus, these results suggest that there is potential for plasticity in the expression of V1aR, and that the expression of nonapeptide receptors in these regions may depend on developmental experiences, pairing status, or other aspects of social context.

Additionally, we found that affiliation with the pair partner was positively correlated with immediate early gene activity in the MeA, including the activity of V1aR expressing cells. This is consistent with evidence that the MeA is involved in affiliative behavior between pair partners in both mammals and songbirds (Kirkpatrick et al., 1994; Svec et al., 2009). These results suggest that the MeA may be similarly involved in pair maintenance behaviors across taxa, perhaps playing a role in both individual recognition and the modulation of contextually-appropriate reproductive behaviors.

Unlike a previous study in zebra finches, we did not find a positive relationship between ZENK-ir in the BSTm and singing (Goodson et al., 2009). Instead, we observed a slight negative correlation between singing and both ZENK-ir in the BSTm, as well as between singing and V1aR+ZENK co-localization in both the BSTm and MeA. In the present study, these samples were not collected from males courting novel females, but instead singing to females with whom they are already paired and in the context of re-establishing or maintaining an already formed pair bond. Heimovics and Riters (2005, 2006) observed differential regulation of starling song by the BSTm depending upon the context in which it is produced. Thus, it is likely that song in this context functions as a pair maintenance behavior, rather than a courtship or sexual behavior, particularly given that no attempted or successful copulations were observed in any of the reunion periods.

AVT has been found to play an important role in vocal behaviors in many species of songbirds, though many details remain poorly understood. The effects of AVT have been found to vary widely depending on the social context, as well as whether the treatments were administered peripherally vs. centrally or acutely vs. chronically (Voorhuis et al., 1991; Maney et al., 1997; Goodson, 1998; Harding and Rowe, 2003). In zebra finches, nonapeptide receptors are expressed in a number of sensorimotor regions where they may influence song production. There is no AVT receptor expression in HVC (a key sensorimotor nucleus in song) and limited expression within the robust nucleus of the arcopallium (RA, homologous to laryngeal motor cortex; Leung et al., 2009, 2011). However, there are high levels AVT immunoreactivity in the intercollicular nucleus (ICo, a region implicated in vocal control) and nXIIts (the key motor nucleus which innervates the syrinx and is considered to be part of the song system).

Previous work in zebra finches and other song birds has demonstrated that adult AVT plays a prominent role in both gregariousness and aggression, but not pairing, particularly in the lateral septum (Kelly et al., 2011). In zebra finches, the LS exhibits a high density of binding of both AVT and OT, as well as high expression of OTR (Leung et al., 2009, 2011; Kelly et al., 2011). Infusions of MC in the LS reduce the preference to affiliate with larger flocks of birds and increase anxiety-like behaviors (Kelly et al., 2011). Consistent with this work, we also failed to find a relationship between activity in the LS and either singing or affiliative behavior, though we did not investigate more generalized sociality.

Indeed, it is possible that housing subjects with a single partner may have obscured many aspects of affiliative and courtship behavior in this species, which normally live in large social flocks. For example, evidence suggests that MC males are more gregarious than either Control or AVT males. In a previous study, these same MC males were found to spend more time than Control or AVT males in proximity with other birds in four-way affiliation tests throughout development, but showed no specific preference to affiliate with female conspecifics (Baran et al., 2016). Because this was a forced-choice pairing experiment, the presence of affiliative behavior observed in the MC males may be a function of preferring to affiliate with other birds in general, rather than the tendency to form a specific pair bond with a female. Furthermore, our paradigm did not allow us to test the effects of treatment on competition between males for female partners.

Nevertheless, our effects were quite specific to the social domain. We observed no effect of the treatment on either feeding or drinking behaviors. We also found no effects of these treatments on growth or survival in chicks or on activity level throughout development (Baran et al., 2016). However, we cannot rule out the possibility that these treatments exerted widespread effects on the hypothalamic-pituitary-adrenal (HPA) axis. AVP/AVT, along with corticotropin releasing factor (CRF), modulates the production of adrenocorticotropic hormone (ACTH) in the anterior pituitary (Buckingham, 2009). Previous work in our lab has shown that separation from the partner results in an elevation of corticosterone, which returns to baseline when individuals are reunited with their pair partner (Remage-Healey et al., 2003). Thus, while there does appear to be a relationship between the HPA axis and pairing, whether the behavioral effects observed in the present study are also partially mediated through alterations to the HPA axis remains to be tested.

In a wide range of socially monogamous species, attachment to parents is observed very early in development, suggesting that the neural substrate underlying affiliative behavior is established long before the formation of adult pair bonds (Bowlby, 1960; Ainsworth, 1989). Furthermore, early experience with caregivers during development is known to influence adult relationships, suggesting that these systems are plastic and sensitive to experience (Bowlby, 1960; Ainsworth, 1989; Champagne et al., 2003; Zayas et al., 2011). From a broader perspective, a deeper understanding of the mechanisms involved in the development of affiliative behaviors is critical for our understanding of how evolution alters developmental processes to create novel behavioral phenotypes (Toth and Robinson, 2007; Hofmann, 2010).

Nonapeptides are among the most evolutionarily labile and context-dependent signaling systems in the vertebrate brain (Goodson, 2005; Choleris et al., 2013). In this experiment, we determined that the nonapeptides play a developmental role in the social brain by injecting large doses of either AVT or MC into the brains of young songbirds. The ability to interpret these effects would be enhanced by descriptive studies of the development of the nonapeptide system in zebra finches. Furthermore, future studies with better spatiotemporal precision will help to elucidate the function of nonapeptides in specific brain regions during development. This would allow researchers to understand how these circuits are contributing to the development of social behaviors, both adaptive and maladaptive. Nevertheless, these results provide strong evidence that the nonapeptides play a critical role in social development and that changes to this system during development can have a profound effect on the social brain throughout life.

## Author contributions

NB conceived of and designed the study, coordinated and carried out all behavioral components, carried out the molecular work in collaboration with MT, performed the statistical analysis, and drafted the manuscript. MT assisted in the design of the study and carried out the molecular work. EA assisted in study design and helped draft the manuscript. All authors gave final approval for publication.

## Funding

This research was supported by an NSF Doctoral Dissertation Improvement Grant (NSF, IOS—1310908); NSF, IOS—1146891; and NIH Training Grant 5T32HD055177-05.

### Conflict of interest statement

The authors declare that the research was conducted in the absence of any commercial or financial relationships that could be construed as a potential conflict of interest.

## References

[B1] Aragona B. J. Liu Y. Yu Y. J. Curtis J. T. Detwiler J. M. Insel T. R.. (2006). Nucleus accumbens dopamine differentially mediates the formation and maintenance of monogamous pair bonds. Nat. Neurosci. 9, 133–139. 10.1038/nn1613 16327783

[B2] Ainsworth M. S. (1989). Attachments beyond infancy. Am. Psychol. 44, 709–716. 10.1037/0003-066X.44.4.709 2729745

[B3] Bales K. L. Carter C. S. (2003a). Developmental exposure to oxytocin facilitates partner preferences in male prairie voles (*Microtus ochrogaster*). Behav. Neurosci. 117, 854–859. 10.1037/0735-7044.117.4.854 12931969

[B4] Bales K. L. Carter C. S. (2003b). Sex differences and developmental effects of oxytocin on aggression and social behavior in prairie voles (*Microtus ochrogaster*). Horm. Behav. 44, 178–184. 10.1016/S0018-506X(03)00154-5 14609540

[B5] Bales K. L. Plotsky P. M. Young L. J. Lim M. M. Grotte N. Ferrer E.. (2007). Neonatal oxytocin manipulations have long-lasting, sexually dimorphic effects on vasopressin receptors. Neuroscience 144, 38–45. 10.1016/j.neuroscience.2006.09.009 17055176 PMC1774559

[B6] Baran N. M. Sklar N. C. Adkins-Regan E. (2016). Developmental effects of vasotocin and nonapeptide receptors on early social attachment and affiliative behavior in the zebra finch. Horm. Behav. 78, 20–31. 10.1016/j.yhbeh.2015.10.005 26476409 PMC4718777

[B7] Bender A. T. Veney S. L. (2008). Treatment with the specific estrogen receptor antagonist ICI 182,780 demasculinizes neuron soma size in the developing zebra finch brain. Brain Res. 1246, 47–53. 10.1016/j.brainres.2008.09.089 18952068

[B8] Boer G. J. (1985). Vasopressin and brain development: studies using the Brattleboro rat. Peptides 6, 49–62. 10.1016/0196-9781(85)90011-7 4047982

[B9] Boer G. J. Quak J. de Vries M. C. Heinsbroek R. P. W. (1994). Mild sustained effects of neonatal vasopressin and oxytocin treatment on brain growth and behavior of the rat. Peptides 15, 229–236. 10.1016/0196-9781(94)90007-8 8008627

[B10] Bowlby J. (1960). Separation anxiety: a critical review of the literature. J. Child Psychol. Psychiatry. 1, 251–269. 10.1111/j.1469-7610.1960.tb01999.x

[B11] Bredewold R. Smith C. J. W. Dumais K. M. Veenema A. H. (2014). Sex-specific modulation of juvenile social play behavior by vasopressin and oxytocin depends on social context. Front. Behav. Neurosci. 8:216. 10.3389/fnbeh.2014.00216 24982623 PMC4058593

[B12] Buckingham J. (2009). Understanding the role of vasopressin in the hypothalamo-pituitary adrenocortical axis, in Perspectives on Vasopressin, ed Laycock J. F. (London: Imperial College Press), 230–256.

[B13] Buijs R. M. Velis D. N. Swaab D. F. (1980). Ontogeny of vasopressin and oxytocin in the fetal rat: early vasopressinergic innervation of the fetal brain. Peptides 1, 315–324. 10.1016/0196-9781(80)90009-1 6892474

[B14] Busnelli M. Bulgheroni E. Manning M. Kleinau G. Chini B. (2013). Selective and potent agonists and antagonists for investigating the role of mouse oxytocin receptors. J. Pharmacol. Exp. Ther. 346, 318–327. 10.1124/jpet.113.202994 23723434 PMC3716315

[B15] Champagne F. A. Francis D. D. Mar A. Meaney M. J. (2003). Variations in maternal care in the rat as a mediating influence for the effects of environment on development. Phys. Behav. 79, 359–371. 10.1016/S0031-9384(03)00149-5 12954431

[B16] Choleris E. Pfaff D. W. Kavaliers M. (eds.). (2013). Oxytocin, Vasopressin and Related Peptides in the Regulation of Behavior. Cambridge: Cambridge University Press.

[B17] Cushing B. S. (2013). The organizational effects of oxytocin and vasopressin, in Oxytocin, Vasopressin and Related Peptides in the Regulation of Behavior, eds Choleris E. Pfaff D. W. Kavaliers M. (Cambridge: Cambridge University Press), 56–72.

[B18] Goodson J. L. (1998). Territorial aggression and dawn song are modulated by septal vasotocin and vasoactive intestinal polypeptide in male field sparrows (*Spizella pusilla*). Horm. Behav. 34, 67–77. 10.1006/hbeh.1998.1467 9735230

[B19] Goodson J. L. (2005). The vertebrate social behavior network: evolutionary themes and variations. Horm. Behav. 48, 11–22. 10.1016/j.yhbeh.2005.02.003 15885690 PMC2570781

[B20] Goodson J. L. Lindberg L. Johnson P. (2004). Effects of central vasotocin and mesotocin manipulations on social behavior in male and female zebra finches. Horm. Behav. 45, 136–143. 10.1016/j.yhbeh.2003.08.006 15019801

[B21] Goodson J. L. Rinaldi J. Kelly A. M. (2009). Vasotocin neurons in the bed nucleus of the stria terminalis preferentially process social information and exhibit properties that dichotomize courting and non-courting phenotypes. Horm. Behav. 55, 197–202. 10.1016/j.yhbeh.2008.10.007 19013174 PMC2652745

[B22] Harding C. F. Rowe S. A. (2003). Vasotocin treatment inhibits courtship in male zebra finches; concomitant androgen treatment inhibits this effect. Horm. Behav. 44, 413–418. 10.1016/j.yhbeh.2003.06.007 14644635

[B23] Heimovics S. A. Riters L. V. (2005). Immediate early gene activity in song control nuclei and brain areas regulating motivation relates positively to singing behavior during, but not outside of, a breeding context. J. Neurobiol. 65, 207–224. 10.1002/neu.20181 16155901

[B24] Heimovics S. A. Riters L. V. (2006). Breeding-context-dependent relationships between song and cFOS labeling within social behavior brain regions in male European starlings (*Sturnus vulgaris*). Horm. Behav. 50, 726–735. 10.1016/j.yhbeh.2006.06.013 16914152 PMC2566848

[B25] Hofmann H. A. (2010). Early developmental patterning sets the stage for brain evolution. Proc. Natl. Acad. Sci. U.S.A. 107, 9919–9920. 10.1073/pnas.1005137107 20508150 PMC2890484

[B26] Immelmann K. (1972). Sexual and other long-term aspects of imprinting in birds and other species. Adv. Study Behav. 4, 147–174. 10.1016/S0065-3454(08)60009-1

[B27] Insel T. R. Young L. J. (2001). The neurobiology of attachment. Nat. Rev. Neurosci. 2, 129–136. 10.1038/35053579 11252992

[B28] Kelly A. M. Goodson J. L. (2014). Hypothalamic oxytocin and vasopressin neurons exert sex-specific effects on pair bonding, gregariousness, and aggression in finches. Proc. Natl. Acad. Sci. U.S.A. 111, 6069–6074. 10.1073/pnas.1322554111 24711411 PMC4000841

[B29] Kelly A. M. Kingsbury M. A. Hoffbuhr K. Schrock S. E. Waxman B. Kabelik D.. (2011). Vasotocin neurons and septal V1a-like receptors potently modulate songbird flocking and responses to novelty. Horm. Behav. 60, 12–21. 10.1016/j.yhbeh.2011.01.012 21295577 PMC3106146

[B30] Kirkpatrick B. Carter C. S. Newman S. W. Insel T. R. (1994). Axon-sparing lesions of the medial nucleus of the amygdala decrease affiliative behaviors in the prairie vole (*Microtus ochrogaster*): behavioral and anatomical specificity. Behav. Neurosci. 108, 501–513. 10.1037/0735-7044.108.3.501 7917044

[B31] Klatt J. D. Goodson J. L. (2013). Oxytocin-like receptors mediate pair bonding in a socially monogamous songbird. Proc. R. Soc. B Biol. Sci. 280:20122396. 10.1098/rspb.2012.2396 23173212 PMC3574448

[B32] Leung C. H. Abebe D. F. Earp S. E. Goode C. T. Grozhik A. V. Mididoddi P.. (2011). Neural distribution of vasotocin receptor mRNA in two species of songbird. Endocrinology 152, 4865–4881. 10.1210/en.2011-1394 22067316 PMC6590851

[B33] Leung C. H. Goode C. T. Young L. J. Maney D. L. (2009). Neural distribution of nonapeptide binding sites in two species of songbird. J. Comp. Neurol. 513, 197–208. 10.1002/cne.21947 19132730

[B34] Lowrey E. M. Tomaszycki M. L. (2014). The formation and maintenance of social relationships increases nonapeptide mRNA in zebra finches of both sexes. Behav. Neurosci. 128, 61–70. 10.1037/a0035416 24512066

[B35] Manning M. Kruszynski M. Bankowski K. Olma A. Lammek B. Cheng L. L.. (1989). Solid-phase synthesis of 16 potent (selective and nonselective) *in vivo* antagonists of oxytocin. J. Med. Chem. 32, 382–391. 10.1021/jm00122a016 2913298

[B36] Manning M. Misicka A. Olma A. Bankowski K. Stoev S. Chini B.. (2012). Oxytocin and vasopressin agonists and antagonists as research tools and potential therapeutics. J. Neuroendocrinol. 24, 609–628. 10.1111/j.1365-2826.2012.02303.x 22375852 PMC3490377

[B37] Maney D. L. Goode C. T. Wingfield J. C. (1997). Intraventricular Infusion of Arginine Vasotocin induces Singing in a Female Songbird. J. Neuroendocrinol. 9, 487–491. 10.1046/j.1365-2826.1997.00635.x 15305566

[B38] Newman S. W. (1999). The medial extended amygdala in male reproductive behavior: a node in the mammalian social behavior network. Ann. N. Y. Acad. Sci. 877, 242–257. 10.1111/j.1749-6632.1999.tb09271.x 10415653

[B39] O'Hara R. B. Kotze D. J. (2010). Do not log-transform count data. Methods Ecol. Evol. 1, 118–122. 10.1111/j.2041-210X.2010.00021.x

[B40] Pedersen A. Tomaszycki M. L. (2012). Oxytocin antagonist treatments alter the formation of pair relationships in zebra finches of both sexes. Horm. Behav. 62, 113–119. 10.1016/j.yhbeh.2012.05.009 22633910

[B41] Pitkow L. J. Sharer C. A. Ren X. Insel T. R. Terwilliger E. F. Young L. J. (2001). Facilitation of affiliation and pair-bond formation by vasopressin receptor gene transfer into the ventral forebrain of a monogamous vole. J. Neurosci. 21, 7392–7396. 11549749 10.1523/JNEUROSCI.21-18-07392.2001 PMC6762997

[B42] Prior N. H. Heimovics S. A. Soma K. K. (2013). Effects of water restriction on reproductive physiology and affiliative behavior in an opportunistically-breeding and monogamous songbird, the zebra finch. Horm. Behav. 63, 462–474. 10.1016/j.yhbeh.2012.12.010 23274698

[B43] Prior N. H. Soma K. K. (2015). Neuroendocrine regulation of long-term pair maintenance in the monogamous zebra finch. Horm. Behav. 76, 11–22. 10.1016/j.yhbeh.2015.04.014 25935729

[B44] Prior N. H. Yap K. N. Soma K. K. (2014). Acute and chronic effects of an aromatase inhibitor on pair-maintenance behavior of water-restricted zebra finch pairs. Gen. Comp. Endocrinol. 196, 62–71. 10.1016/j.ygcen.2013.10.018 24231681

[B45] R Core Team (2015). R: A Language and Environment for Statistical Computing, Vienna: R Foundation for Statistical Computing. Available online at: https://www.R-project.org/

[B46] Remage-Healey L. Adkins-Regan E. Romero L. M. (2003). Behavioral and adrenocortical responses to mate separation and reunion in the zebra finch. Horm. Behav. 43, 108–114. 10.1016/S0018-506X(02)00012-0 12614640

[B47] Stribley J. M. Carter C. S. (1999). Developmental exposure to vasopressin increases aggression in adult prairie voles. Proc. Natl. Acad. Sci. U.S.A. 96, 12601–12604. 10.1073/pnas.96.22.12601 10535968 PMC23008

[B48] Svec L. A. Licht K. M. Wade J. (2009). Pair bonding in the female zebra finch: a potential role for the nucleus taeniae. Neuroscience 160, 275–283. 10.1016/j.neuroscience.2009.02.003 19409212 PMC2684567

[B49] Szot P. Dorsa D. M. (1993). Differential timing and sexual dimorphism in the expression of the vasopressin gene in the developing rat brain. Dev. Brain Res. 73, 177–183. 10.1016/0165-3806(93)90136-X 8353930

[B50] Toth A. L. Robinson G. E. (2007). Evo-devo and the evolution of social behavior. Trends Genet. 23, 334–341. 10.1016/j.tig.2007.05.001 17509723

[B51] Veenema A. H. Bredewold R. De Vries G. J. (2012). Vasopressin regulates social recognition in juvenile and adult rats of both sexes, but in sex- and age-specific ways. Horm. Behav. 61, 50–56. 10.1016/j.yhbeh.2011.10.002 22033278 PMC3264802

[B52] Voorhuis T. A. M. de Kloet E. R. de Wied D. (1988). The distribution and plasticity of [3H]vasopressin-labelled specific binding sites in the canary brain. Brain Res. 457, 148–153. 10.1016/0006-8993(88)90067-4 2971421

[B53] Voorhuis T. A. M. De Kloet E. R. De Wied D. (1991). Effect of a vasotocin analog on singing behavior in the canary. Horm. Behav. 25, 549–559. 10.1016/0018-506X(91)90020-I 1813380

[B54] Winslow J. T. Hastings N. Carter C. S. Harbaugh C. R. Insel T. R. (1993). A role for central vasopressin in pair bonding in monogamous prairie voles. Nature 365, 545–548. 10.1038/365545a0 8413608

[B55] Winslow J. T. Insel T. R. (1993). Effects of central vasopressin administration to infant rats. Eur. J. Pharmacol. 233, 101–107. 10.1016/0014-2999(93)90354-K 8472738

[B56] Yamamoto Y. Cushing B. Kramer K. Epperson P. Hoffman G. Carter C. (2004). Neonatal manipulations of oxytocin alter expression of oxytocin and vasopressin immunoreactive cells in the paraventricular nucleus of the hypothalamus in a gender-specific manner. Neuroscience 125, 947–955. 10.1016/j.neuroscience.2004.02.028 15120854

[B57] Zann R. A. (1996). The Zebra Finch: A Synthesis of Field and Laboratory Studies. Oxford, UK: Oxford University Press.

[B58] Zayas V. Mischel W. Shoda Y. Aber J. L. (2011). Roots of adult attachment maternal caregiving at 18 months predicts adult peer and partner attachment. Soc. Psychol. Person. Sci. 2, 289–297. 10.1177/1948550610389822

