# Supplementary material for: Early Life Manipulations of the Nonapeptide System Alter Pair Maintenance Behaviors and Neural Activity in Adult Male Zebra Finches
Source: Front Behav Neurosci. 2016 Mar 29;10:58. doi: 10.3389/fnbeh.2016.00058 (PMC4810809; doi:10.3389/fnbeh.2016.00058)
Supplement: Supplementary file 2 [file Presentation1.PDF]

Supplementary Material

Early Life Manipulations of the Nonapeptide System Alter Pair Maintenance Behaviors and Neural Activity in Adult Male Zebra Finches

Nicole M. Baran1,2*, Michelle L. Tomaszycki3,4, Elizabeth Adkins-Regan1,5

[1] Department of Psychology, Cornell University, Ithaca, NY
[2] School of Biology, Georgia Institute of Technology, Atlanta, GA
[3] Department of Psychology, Wayne State University, Detroit, MI
[4] Department of Psychology, Lafayette College, Easton, PA
[5] Department of Neurobiology & Behavior, Cornell University, Ithaca, NY

***Correspondence:** Corresponding Author: nicole.baran@biology.gatech.edu

1 Supplementary Video 1: Reunion behavior of Control and AVT male

This video depicts the reunions between a Control male (on left) and an AVT male (on right) and their female partners. These reunions occurred at the same time and were filmed side-by-side. First, a research assistant releases the female partner of the Control male into the aviary (00:02). The Control male can be observed singing to the female within seconds (00:05). Then the female partner of the AVT male is released into the aviary (00:12). The AVT male also begins singing very quickly (00:17) and there is a brief bout of beak fencing at 00:20. At 00:24, the video skips ahead approximately 3 minutes. The AVT male can be observed initiating a bout of clumping with the female at 00:26. The AVT male was observed to be perched in contact with his partner for a total of 7.5 min of the 25 min reunion period. In contrast, the Control male is not observed perching in contact with the female at all during the reunion period. Birds in other aviaries can also be heard singing in the background.
